# Cell-specific *MAPT* gene expression is preserved in neuronal and glial tau cytopathologies in progressive supranuclear palsy

**DOI:** 10.1007/s00401-023-02604-x

**Published:** 2023-06-24

**Authors:** Shelley L. Forrest, Seojin Lee, Nasna Nassir, Ivan Martinez-Valbuena, Valerie Sackmann, Jun Li, Awab Ahmed, Maria Carmela Tartaglia, Lars M. Ittner, Anthony E. Lang, Mohammed Uddin, Gabor G. Kovacs

**Affiliations:** 1 Tanz Centre for Research in Neurodegenerative Disease (CRND), University of Toronto, Krembil Discovery Tower, 60 Leonard Ave, Toronto, ON M5T 0S8 Canada; 2 Dementia Research Centre, Macquarie Medical School, Faculty of Medicine, Health and Human Sciences, Macquarie University, Sydney, Australia; 3 Laboratory Medicine Program and Krembil Brain Institute, University Health Network, Toronto, ON Canada; 4 College of Medicine, Mohammed Bin Rashid University of Medicine and Health Sciences, Dubai, UAE; 5 University Health Network Memory Clinic, Krembil Brain Institute, Toronto, ON Canada; 6 Edmond J. Safra Program in Parkinson’s Disease, Rossy PSP Centre and the Morton and Gloria Shulman Movement Disorders Clinic, Toronto Western Hospital, Toronto, ON Canada; 7 Cellular Intelligence (Ci) Lab, GenomeArc Inc., Toronto, ON Canada; 8 Department of Laboratory Medicine and Pathobiology and Department of Medicine, University of Toronto, Toronto, ON Canada

**Keywords:** Astrocyte, *MAPT*, Oligodendrocyte, Progressive supranuclear palsy, RNA, Tau

## Abstract

Microtubule-associated protein tau (*MAPT*) aggregates in neurons, astrocytes and oligodendrocytes in a number of neurodegenerative diseases, including progressive supranuclear palsy (PSP). Tau is a target of therapy and the strategy includes either the elimination of pathological tau aggregates or reducing *MAPT* expression, and thus the amount of tau protein made to prevent its aggregation. Disease-associated tau affects brain regions in a sequential manner that includes cell-to-cell spreading. Involvement of glial cells that show tau aggregates is interpreted as glial cells taking up misfolded tau assuming that glial cells do not express enough *MAPT*. Although studies have evaluated *MAPT* expression in human brain tissue homogenates, it is not clear whether *MAPT* expression is compromised in cells accumulating pathological tau. To address these perplexing aspects of disease pathogenesis, this study used RNAscope combined with immunofluorescence (AT8), and single-nuclear(sn) RNAseq to systematically map and quantify *MAPT* expression dynamics across different cell types and brain regions in controls (*n* = 3) and evaluated whether tau cytopathology affects *MAPT* expression in PSP (*n* = 3). *MAPT* transcripts were detected in neurons, astrocytes and oligodendrocytes, and varied between brain regions and within each cell type, and were preserved in all cell types with tau aggregates in PSP. These results propose a complex scenario in all cell types, where, in addition to the ingested misfolded tau, the preserved cellular *MAPT* expression provides a pool for local protein production that can (1) be phosphorylated and aggregated, or (2) feed the seeding of ingested misfolded tau by providing physiological tau, both accentuating the pathological process. Since tau cytopathology does not compromise *MAPT* gene expression in PSP, a complete loss of tau protein expression as an early pathogenic component is less likely. These observations provide rationale for a dual approach to therapy by decreasing cellular *MAPT* expression and targeting removal of misfolded tau.

**Supplementary Information:**

The online version contains supplementary material available at 10.1007/s00401-023-02604-x.

## Introduction

Tau is a microtubule-associated protein encoded by the *MAPT* gene, which was initially described as a neuronal protein that is enriched in axons and has important functions in binding and stabilizing the cytoskeleton and regulating cellular transport [26, 68]. Recent studies also highlight that tau interacts with other cellular structures including cytoplasmic organelles, plasma membrane, the actin cytoskeleton, nucleus and dendrites [2, 8, 30, 32, 61, 62]. Tau is the most commonly deposited protein in the ageing brain and in neurodegenerative diseases, including progressive supranuclear palsy (PSP), where it aggregates in neurons, oligodendrocytes and astrocytes [16, 17]. The hallmark pathological features of PSP include neurofibrillary tangles (NFTs) in neurons in subcortical and brainstem regions in combination with tau-immunopositive tufted astrocytes [56]. Tau-immunopositive oligodendrocytic coiled bodies are also a common and widespread neuropathological feature of PSP. Tau is a major therapeutic target for PSP and other tau-depositing neurodegenerative diseases, which are currently focused on the elimination of pathological aggregates from the brain or directly targeting *MAPT* gene expression with antisense oligonucleotide (ASO) therapies to inhibit tau aggregation [5, 34, 52, 57, 63].

Disease-associated tau affects brain regions in a hierarchical and sequential manner that includes cell-to-cell spreading, which is well described for neurons [20, 57, 67]. Involvement of glial cells showing fibrillar tau aggregates has been interpreted as indicating that glial cells internalize disease-associated tau released from neurons [35]. These concepts assume that glial cells do not express substantial amounts of *MAPT*. Although several studies have evaluated *MAPT* expression in tissue homogenates [6, 24, 29, 47, 65, 66], in humans there is a paucity of studies focusing on *MAPT* gene expression in glia. Early in situ hybridization studies that mapped the distribution of tau mRNA in the cerebral cortex and hippocampus of adult human brain showed that tau mRNA is expressed in neurons and is absent in glia [23]. However, oligodendrocytes have an elaborate microtubule network, which provide the tracks for organelle trafficking and intracellular translocation of myelin gene products, suggesting that tau may have functions in regulating microtubule-dependent processes as it does in neurons [31]. Low levels of tau mRNA have been identified in human oligodendrocytes (http://www.brainrnaseq.org) [71] and low levels of *MAPT* expression in cultured oligodendrocytes [45, 46, 51, 58], as well as astrocytes and oligodendrocytes in online databases [48]. A recent study that used tauGFP knock-in/knock-out mice with an eGFP-coding sequence inserted into the first exon of the* MAPT* gene generating cytoplasmic eGFP expression under the endogenous tau promoter reported endogenous expression only in oligodendrocytes and neurons [66]. These observations are consistent with the detection of tau protein in oligodendrocytes using Tau5 immunohistochemistry [46]. In contrast, limited studies suggest that tau might be expressed in astrocytes. For example, tau mRNA is expressed in low amounts in astrocytes (http://www.brainrnaseq.org) [71] and has been reported in astrocytic tumours [49]. Tau-immunostaining has also been reported in astrocytes in the normal human brain and in astrocytic tumours [49, 59]. Despite these studies, the prevailing view is that glia do not express or contain sufficient amounts of *MAPT* mRNA and tau protein to build fibrillar inclusions independently in response to an unidentified neurodegeneration-inducing event in PSP and other tau-related neurodegenerative diseases [35, 38].

A number of neuropathological studies in humans have reported the presence of astrocytic or oligodendrocytic tau-immunopositive inclusions in the absence of neuronal tau [18, 37]. Further observations on the distinct sequential distribution of glial tau cytopathologies also raise the possibility of whether these can develop independently of neuronal tau [40, 41]. Post-translational modifications of tau have been examined in astrocytes and oligodendrocytes and show that tau in these cell types is phosphorylated at different sites and has an altered conformation [14]. These observations could suggest that glial cells have basic *MAPT* and tau protein expression that can be phosphorylated and fibrillized into a pathological inclusion, rather than tau being taken up from neurons.

Another uncertain aspect of disease pathogenesis is whether *MAPT* expression is compromised in cells accumulating phosphorylated pathological tau aggregates (i.e. a “loss-of-function” component to the pathogenesis). To address these perplexing aspects of disease pathogenesis, this study used RNAscope combined with immunostaining, complemented by single-nucleus (sn)RNA sequencing from PSP and control human brains, to systematically map and quantify *MAPT* expression in neurons, oligodendrocytes and astrocytes across different brain regions to determine whether there are cell type and/or regional differences in *MAPT* expression. This study also investigated whether *MAPT* gene expression is altered in neurons or glia containing tau-immunopositive inclusions in PSP.

## Materials and methods

### Cases and tissue collection

Brains from three control individuals without neurodegenerative pathology (3 male; mean age: 75 years) and three PSP patients who underwent medical assistance in dying (MAID; 3 male; mean age: 75 years) were included in this study. Participants were prospectively enrolled in longitudinal multidisciplinary research programmes and recruited with informed consent through local brain donor programmes. Demographic and genetic (*MAPT* haplotype) information was collected from an integrated clinicopathological database. All cases included in this study had a routine neuropathological assessment using standardized neuropathological consensus recommendations. The three control cases were selected based on their similar postmortem delay (4–12.5 h) to the PSP cases (3.5–14 h) included in this study.

### RNAscope combined with immunostaining for AT8

To map the anatomical distribution of *MAPT* gene expression in different cell types throughout the brain, formalin-fixed paraffin-embedded 4 μm sections from the superior frontal cortex, hippocampus, striatum, midbrain and cerebellum from one control case were processed for RNAscope combined with AT8 immunoperoxidase and for RNAscope combined with AT8 immunofluorescence. To determine *MAPT* gene expression in cellular cytopathologies, formalin-fixed paraffin-embedded 4 μm sections from the superior frontal cortex and striatum from three PSP cases were processed for RNAscope combined with AT8 immunofluorescence. Sections were deparaffinized and rehydrated to perform the following RNAscope^®^ in situ hybridization assay according to the manufacturer’s instructions (Advance Cell Diagnostics (ACD), Hayward, CA, USA). Sections were incubated with the RNAscope^®^ hydrogen peroxide for 10 min at RT and were pre-treated in boiling RNAscope^®^ 1X Target Retrieval Reagents solution for 15 min. Sections were then incubated in the RNAscope^®^ Protease Plus solution for 30 min at 40 °C. Each section was hybridized with the *MAPT* probe (408991-C2) and one of the following cell type-specific probes: *Olig2* (424191-C1), *RBFOX3* (415591-C4) and *ALDH1L1* (438881-C3), for 2 h at 40 °C. Signals were amplified using RNAScope® Multiplex FL v2 Amp 1,2, and 3 according to the manufacturer’s directions. Signals were then developed accordingly to the appropriate channels. Opal 570 dye (1:1500; Akoya Biosciences, Marlborough, MA, USA) was used to visualize *MAPT* probes and Opal 690 dye (1:1000) was used for visualization of the cell type-specific probes. Following a final wash, sections were proceeded to immunofluorescence staining. Briefly, sections were washed with DAKO washing buffer (Dako, Santa Clara, CA, USA), blocked with DAKO peroxidase solution for 10 min and incubated with anti-tau AT8 (Phospho-Tau, Ser202, Thr205; 1:1000; Thermo Fisher) for 1 h at RT, followed by the secondary antibody Alexa Fluor 488 donkey anti-mouse IgG (H + L) (1:500; Thermo Fisher, Rockford, IL, USA) for 1 h at RT in the dark. All sections were counterstained with DAPI to visualize cell nuclei and mounted with ProLong^™^ Gold Antifade Mountant (Thermo Fisher, Rockford, IL, USA). A similar protocol was used for RNAscope for light microscopy based on manufacturer’s instructions, using the same *MAPT*, *RBFOX3*, *Olig2* and *ALDH1L1* probes as above (https://acdbio.com/sites/default/files/RNAscope_Sample_Preparation_Pretreatment_Guide_FFPETissue.pdf), omitting AT8 immunostaining.

### Morphometry

Images of RNAscope fluorescent sections were analysed using algorithms created on the General Analysis 3 module in NIS Elements software (version 5.30.04, Nikon Instruments Inc.). Cell types of interest were identified by capturing nuclei (DAPI) which were then extended in radius length corresponding to the size of cell types in the spatially defined regions and annotating those which contained positivity for cell type marker (*RBFOX3*, *Olig2*, *ALDH1L1*) probes within the defined cell area. Cell marker positivity was thresholded by fluorescence intensity and area to eliminate false detection in different cell types due to extending processes or proximity among cells. In the substantia nigra, cerebellum (Purkinje cells) and globus pallidus, neurons were manually annotated due to their distinct morphologies in respect to the nucleus. Annotated cells were converted into individual regions of interest for evaluation of *MAPT* transcripts in each cell. *MAPT* transcripts were observed individually or in small confluent clusters and for this reason, individual transcripts could not be counted with confidence. Therefore, in  this study, we used the area density values of *MAPT* transcripts. This was calculated by the total area of *MAPT* transcripts divided by the annotated area for each cell and expressed as a percentage. To measure nuclear area density of *MAPT* transcripts in oligodendrocytes and astrocytes, a region of interest was manually drawn around the nucleus, identified with DAPI, in oligodendrocytes and astrocytes without tau-immunopositive inclusions in the basal ganglia of three PSP cases.

### Single-nuclear (sn)RNAseq

Tissue was collected from the frontal cortex in three control and three PSP cases and flash frozen and stored at −80C for snRNAseq. Frozen brain tissue was chopped into smaller pieces on dry ice. Frozen tissues were immediately loaded on the Singulator TM 100 (S2 Genomics1). Nuclei isolation was operated by following the “Small Volume Nuclei Isolation Protocol” from the Automated Tissue Dissociation System with minor adjustments. The Singulator 100 instrument was primed with cold nuclei isolation (NIS) and storage buffers (NSR). Frozen tissues were loaded on the Singulator cartridge which was primed with a 40 µm filter. The cartridge was immediately mounted onto the system for nuclei isolation. All of the above materials were supplied by S2 Genomics. In addition, RNase inhibitor (Sigma-Aldrich; Cat # 3335402001) was added to the Singulator Cartridge to reach a final concentration of 0.2U/μl. Nuclei collected from the Singulator was spun at 800×*g*, at 4 °C for 10 min. The supernatant was removed, followed by resuspending nuclei in freshly made cold wash and resuspension buffer (1× PBS, 1% BSA, 0.2U/μl). The wash was repeated twice. Nuclei were stained with DAPI and sorted for DAPI-positive cells to exclude any debris or nuclei aggregates using an Aria Fusion A cell sorter. The nuclei were collected in wash and resuspension buffer and spun at 800×*g* for 10 min at 4 °C. The supernatant was removed, and then resuspended in wash and resuspension buffer. Nuclei were stained with SYBR Green II and counted under a microscope using INCYTO C-Chip Hemocytometer (Neubauer Improved).

Sorted nuclei were used as input into the 10× Genomics single-cell 3′ v3.1 assay and processed as described by the protocol provided by the 10X Genomics. Library construction and library sequencing proceeded as described in the 10X Genomics protocol. In brief, the molarity of each library was calculated based on library size as measured by bioanalyser (Agilent Technologies) and qPCR amplification data (Roche). Samples were pooled and normalized to 1.5 nM. Library pool was denatured using 0.2N NaOH (Sigma) for 8 min at room temperature and neutralized with 400 mM Tris–HCL (Sigma). Library pool at a final concentration of 300 pM were loaded to sequence on Novaseq 6000 (Illumina). Samples were sequenced with the following run parameters: Read 1–28 cycles, Read 2–90, index 1–10 cycles, index 2–10 cycles. Sequencing target read depth per library was ~ 40,000 reads per nuclei. snRNAseq was performed by the Princess Margaret Genomics Centre (Toronto, Canada).

### Alignment, barcode assignment and UMI counting

Single-nuclei transcriptome sequencing data were pre-processed using the Cell Ranger Single-Cell Software Suite (v 6.0.0) (10X Genomics). Cell barcodes and UMI (unique molecular identifiers) barcodes were demultiplexed and single-end reads aligned to the reference genome, GRCh38, using the STAR (v 2.7.10) [11] tool of Cell Ranger pipeline. The resulting cell–gene matrix contains UMI counts by gene and by cell barcode. To proceed with the clustering analysis, three each (control, PSP) gene expression quantification experiments were merged into a single- feature-barcode matrix. ‘CellRanger aggr’ runs depth normalization algorithm to make all merged datasets have a similar number of uniquely mapped transcriptome reads per cell.

### Clustering and differential gene expression analysis

Dimensionality reduction was performed by running principal component analysis (PCA). Ten PCs were retained for the analysis and cells were clustered based on k-means algorithm, visualized using UMAP (uniform manifold approximation and projection). We assessed cluster-specific marker genes in PSP and control clusters by detecting the differentially expressed genes between the given cluster and the other clusters (*p* < 0.05; negative binomial exact test).

### Cell type annotation

The cell types were annotated by mapping the known brain cell type markers [28] to the differentially expressed marker genes in each cluster. We employed stringent criteria where three approaches were used to assign cell type identity to each cluster [53, 54]. First, we plotted the number of overlapping genes in known marker genes and cluster differentially expressed genes (top 50 genes in each cluster, based on log2 fold change). Second, we plotted the mean expression of differentially expressed marker genes across all clusters. Third, we quantified the mean expression of the known marker genes across clusters. Heatmaps and boxplots were generated using the plotly package in Python. Cell type identity was assigned to each cluster based on the restricted expression of marker genes (combining three approaches).

### MAPT gene expression

Control and PSP expression matrices were normalized to 10,000 reads per cell and transformed using log2 (expression) using Scanpy (v 1.7.2). These values were used to quantify *MAPT* expression across different cell types in controls and PSP.

### Statistical evaluation

SPSS Statistics version 23 and Prism (version 9.5.1) were used for statistical analyses. Mann–Whitney *U* tests were used to compare the difference in *MAPT* gene expression between control and PSP groups in snRNAseq studies. For RNAscope studies, normality of the area density of *MAPT* gene transcripts in neurons, oligodendrocytes and astrocytes with and without tau-immunopositive inclusions was tested using the Kolmogorov–Smirnov test [10] and *p* values < 0.05 were regarded as having a non-normal distribution. Mann–Whitney *U* and Kruskal–Wallis tests were used to compare the area density of *MAPT* gene transcripts in neurons, oligodendrocytes and astrocytes with and without tau-immunopositive inclusions, and between groups. The standard significance level was set at 0.01. The z-score (standard score) cutoff value was used as an additional conservative approach [10] to determine the proportion (%) of cells with tau-immunopositive inclusions with area density values above or below the 95% percentile of the area density values of cells without tau-immunopositive inclusions. If this proportion reached 50% of cells, we interpret this as a strong indicator for an increase or decrease of *MAPT* transcript area density compared with cells without tau-immunopositive inclusions. Area density is reported as the mean ± standard error.

## Results

### Demographics and characterization of cases examined

Demographic and neuropathological data of patients used in this study are summarized in Table 1. Controls ranged from 74 to 77 years at death, and PSP cases ranged from 73 to 77 years at death. All cases used in this study had an absence or low AD neuropathological change. All PSP cases were *H1/H1 MAPT* haplotype.

**Table 1:** Demographic and neuropathological data of patients

Diagnosis	Age at death (y)	Disease duration (y)	Sex	PMI (*h*)	Cause of death	ADNC [50]	PSP stage [41]
Control 1	74	–	M	12.5	Aortic aneurysm	None	0
Control 2	74	–	M	6.25	Heart failure	None	0
Control 3	77	–	M	4	Myocardial infarction	None	0
PSP 1	76	5	M	14	MAID	None	3
PSP 2	73	3	M	3.5	MAID	None	4
PSP 3	77	8	M	4.5	MAID	Low	4

*ADNC* Alzheimer’s disease neuropathological change; *MAID* medical assistance in dying; *PMI* postmortem interval; *PSP* progressive supranuclear palsy

### Light microscopy RNAscope

**Fig. 1:**
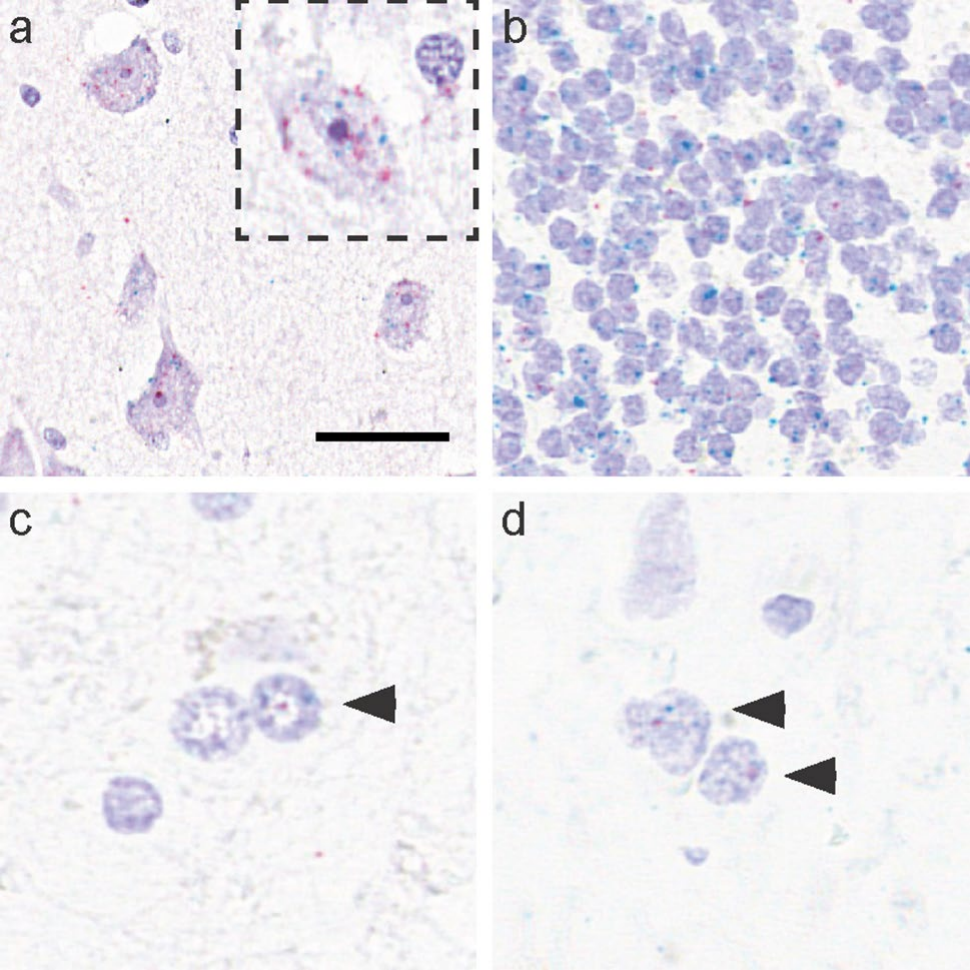
*MAPT* transcripts in neurons and glia revealed by light microscopy RNAscope in a control case. **a** *MAPT* transcripts (red) in neurons in the frontal cortex are observed individually or in small clusters of transcripts. *MAPT* transcripts were found in both the nucleus and cytoplasm. Neurons contain *RBFOX3* transcripts (blue), the cell-specific marker. Dashed box in (**a**) shows a pyramidal neuron at higher magnification. **b** *MAPT* (red) and *RBFOX3* (blue) transcripts in cerebellar granular neurons. **c** *MAPT* transcripts (red) in oligodendrocytes were commonly located in the nucleus, shown with oligodendrocyte-specific transcripts, *Olig2* (blue). **d** *MAPT* transcripts (red) in astrocytes were also commonly found in the nucleus, shown with astrocyte-specific transcripts, *ALDH1L1* (blue). Scale bar in (**a**) represents 30 μm for (**a**) (inset 50 μm), 50 μm for (**b**), 10 μm for (**c**) and 8 μm for (**d**)

*MAPT* transcripts revealed by RNAscope in controls showed expression in neurons, oligodendrocytes and astrocytes in all brain regions examined (Fig. 1). The cell-specific transcripts, *RBFOX3*, *Olig2* and *ALDH1L1*, were observed in the nuclei and cytoplasm of neurons, oligodendrocytes and astrocytes, respectively. In neurons, *MAPT* transcripts were observed in both the nucleus and cytoplasm, individually or in clusters of transcripts (Fig. 1a, b). In oligodendrocytes and astrocytes, *MAPT* transcripts were observed as individual transcripts and located in the nucleus and cytoplasm (Fig. 1c, d).

### Fluorescence RNAscope combined with immunostaining for AT8

**Fig. 2:**
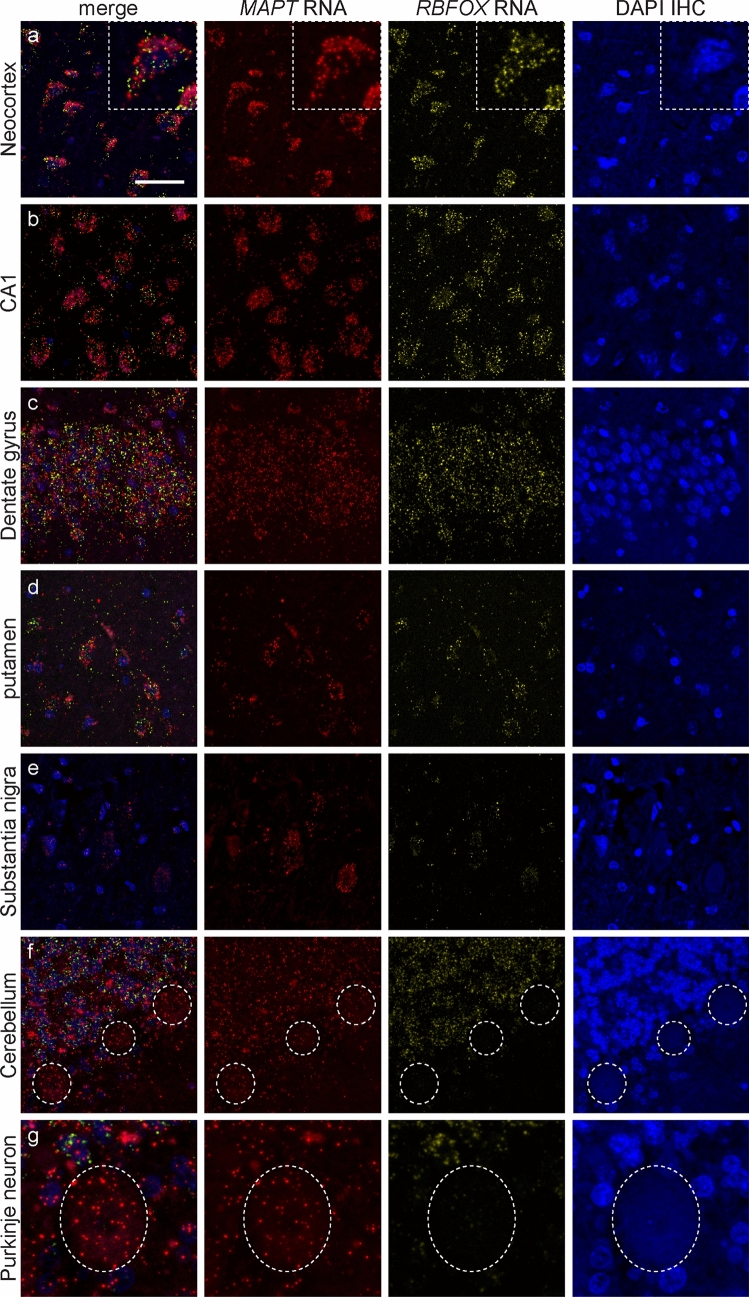
*MAPT* transcripts revealed by fluorescent RNAscope in neurons in a control case. Each horizontal set represents confocal images taken from the same field of view showing *MAPT* (red) and the neuron-specific *RBFOX3* (yellow) transcripts, and the merged image. Sections counterstained with DAPI (blue) to identify nuclei. *MAPT* transcripts were found in the nucleus and cytoplasm of neurons and the number of transcripts varied between neurons in all regions examined including the neocortex (**a**), hippocampal CA1 region (**b**) and dentate gyrus (**c**), the putamen (**d**), substantia nigra (**e**), cerebellar granular (**f**) and Purkinje neurons (**g**). Dashed box in (**a**) shows a pyramidal neuron at higher magnification. Dashed circles in (**f**) show the location of Purkinje neurons, enlarged in (**g**). A high density of *MAPT* and *RBFOX3* transcripts was found in the hippocampal dentate gyrus (**c**) and cerebellar granular neurons (**f**), corresponding to the high density of neurons in these areas. Scale bar in (**a**) represents 30 μm for (**a**) (inset 10 μm) and applies to (**b**, **d**, **e**), 40 μm for (**c**), 50 μm for (**f**), and 20 μm for (**g**)

**Fig. 3:**
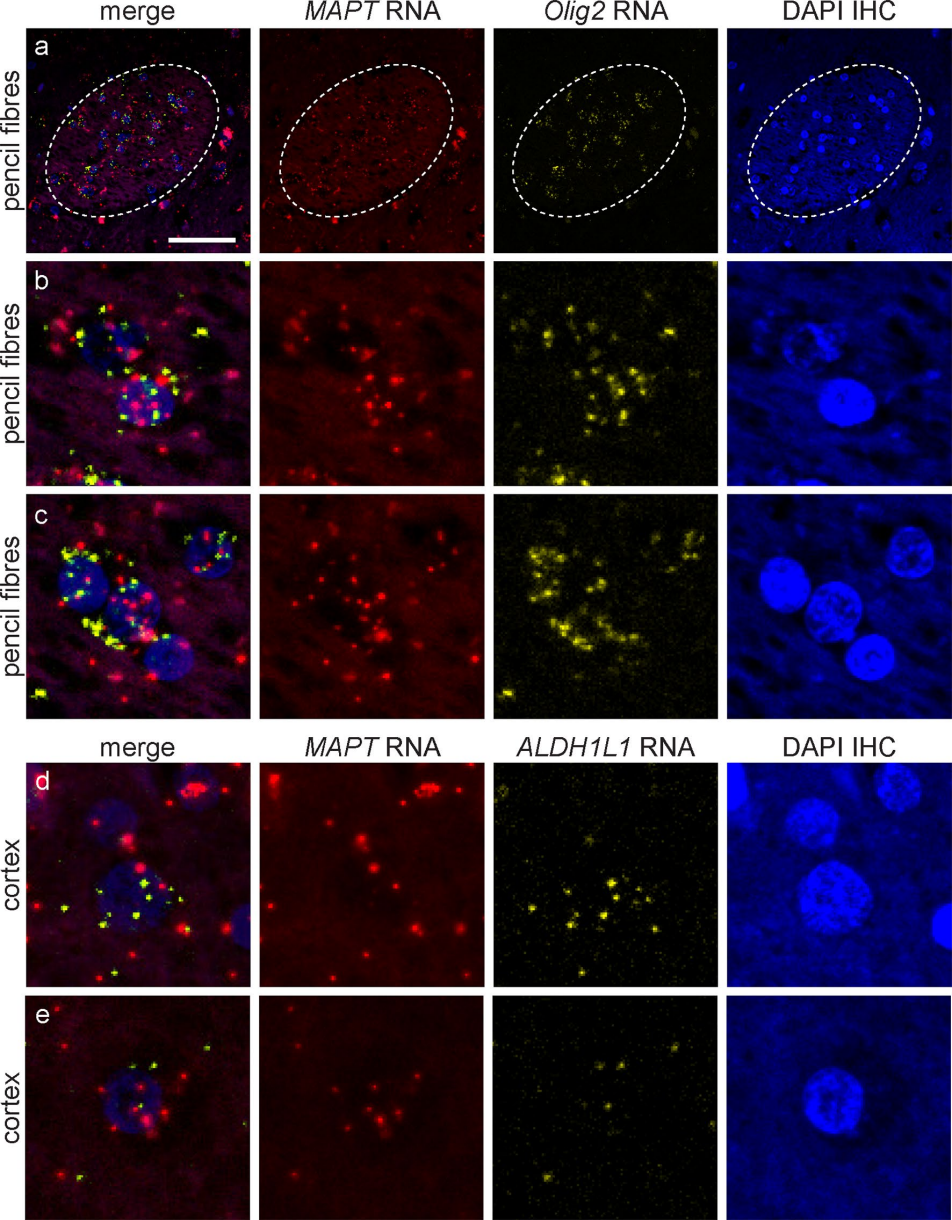
*MAPT* transcripts revealed by fluorescent RNAscope in oligodendrocytes and astrocytes in a control case. Each horizontal set represents confocal images taken from the same field of view showing *MAPT* (red) and the oligodendrocyte-specific *Olig2* (yellow) or the astrocyte-specific *ALDH1L1* (yellow) transcripts, DAPI (blue) and the merged image. *MAPT* transcripts were found in the cytoplasm and nucleus of oligodendrocytes in all brain regions examined including pencil fibres of Wilson (**a**), enlarged in panels (**b** and **c**). A high density of *MAPT* and *Olig2* transcripts were found in pencil fibres of Wilson, corresponding to the high density of oligodendrocytes in these fibre bundles. The number of *MAPT* transcripts in oligodendrocytes varied (**b**, **c**). *MAPT* transcripts were also observed in astrocytes in all brain regions examined including the neocortex (**d**, **e**). Similar to oligodendrocytes, the number of *MAPT* transcripts in astrocytes varied (**d**, **e**). Scale bar in (**a**) represents 30 μm for (**a**), 8 μm for (**b**, **c**, **d**) and 15 μm for (**e**)

In the control case, similar to the light microscopy RNAscope, *MAPT* transcripts revealed by fluorescent RNAscope were observed in neurons, oligodendrocytes and astrocytes in all brain regions examined as confirmed with the cell-specific transcripts, *RBFOX3*, *Olig2* and *ALDH1L1*, respectively (Figs. 2 and 3). Within each cell type, a similar pattern of *MAPT* transcripts was observed in the cytoplasm and nucleus as described above. *MAPT* transcripts varied within neurons, oligodendrocytes and astrocytes, and between brain regions for each cell type. *RBFOX3* transcripts predominated in the cytoplasm compared to the nucleus of neurons. A high density of *RBFOX3* transcripts was observed in the hippocampal dentate gyrus and cerebellar granule cell layer, corresponding to the high density of neurons in these regions (Fig. 2c, f). *Olig2* and *ALDH1L1* transcripts in oligodendrocytes and astrocytes (Fig. 3), respectively, were found in the cytoplasm and nucleus. Distinct islands with a high density of *Olig2* transcripts were observed in the pencil fibres of Wilson, corresponding to the high density of oligodendrocytes in these fibre bundles compared with the surrounding grey matter (Fig. 3a). *MAPT* transcripts were not detected in the Schwann cells of the extraneural segment of the oculomotor nerve and only occasional *MAPT* transcripts were seen in the axons of the oculomotor nerve (Online Resource 1). Importantly, *MAPT*, *RBFOX3*, *Olig2* and *ALDH1L1* transcripts were not observed in endothelial cells (Online Resource 2). AT8-immuopositive inclusions were not observed in the control case.

**Fig. 4:**
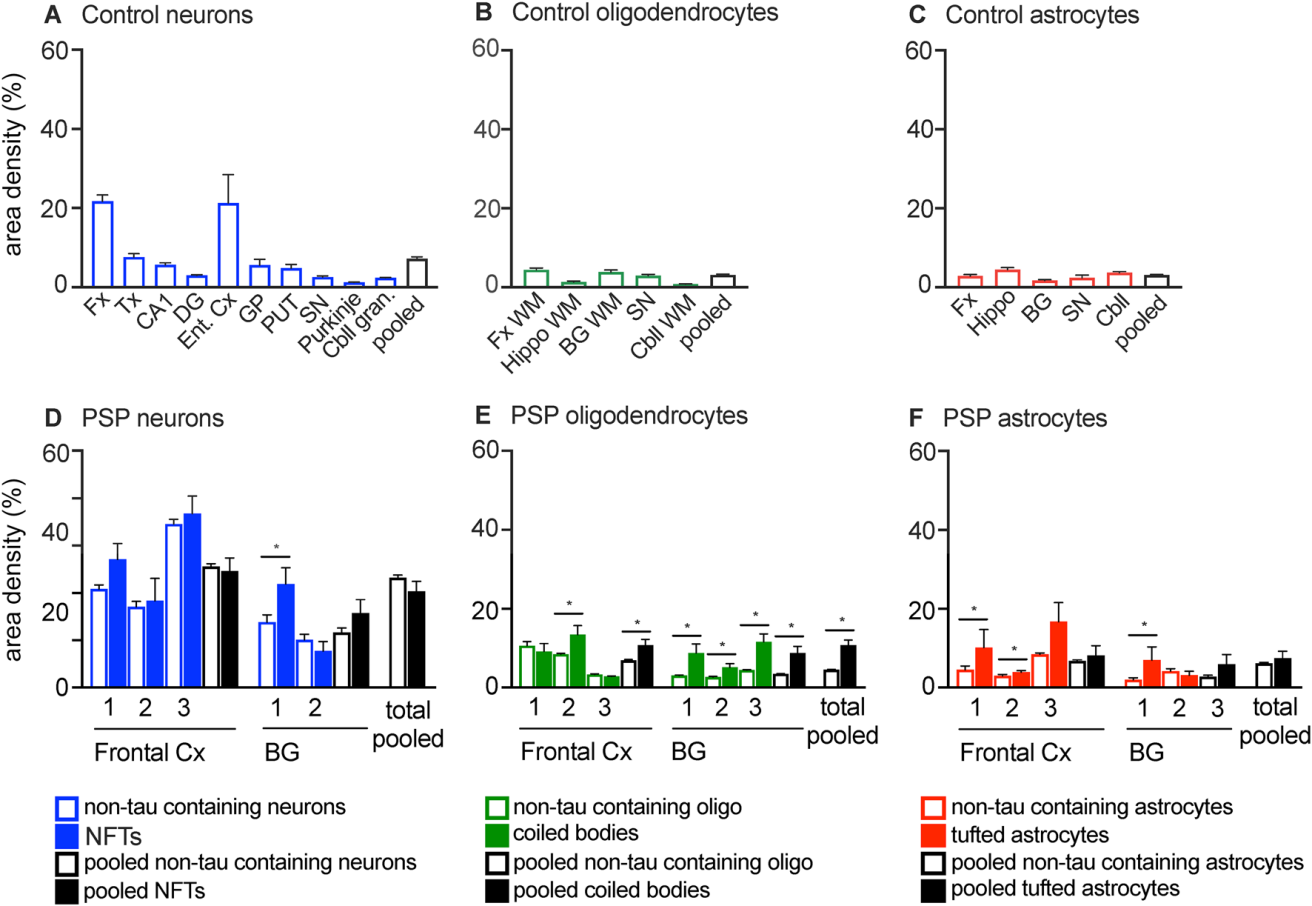
Area density (%) of *MAPT* transcripts in control and PSP cases. Area density of *MAPT* transcripts in neurons (**a**), oligodendrocytes (**b**) and astrocytes (**c**) in a control case in each brain region examined. The black outlined bar in each panel (**a**–**f**) represents the pooled mean area density from each brain region for that cell type, and in panels (**d**–**f**) the solid black bars represent cells with tau-immunopositive inclusions. Numbers along the x-axis in (**d**–**f**) represent the three PSP cases in each region. **d** Similar area density of *MAPT* transcripts was found in neurons without tau-immunopositive inclusions (blue outline) and in neurons containing neurofibrillary tangles (solid blue bars) in the three PSP cases in the frontal cortex and basal ganglia, and pooled from each region and both brain regions. **e** Area density of *MAPT* transcripts in oligodendrocytes without tau-immunopositive inclusions (green outline) and in oligodendrocytic coiled bodies (solid green). The area density of *MAPT* transcripts was increased in oligodendrocytic coiled bodies compared with oligodendrocytes without inclusions (*p* < 0.001) in all cases in the basal ganglia and in the pooled oligodendrocytes. **f** Area density of *MAPT* transcripts in astrocytes without tau-immunopositive inclusions (red outline) and tufted astrocytes (solid red). An increased area density of *MAPT* transcripts was observed in some cases (*p* < 0.001). *BG* basal ganglia; *CA1* hippocampal CA1 region; *Cbll* cerebellum; *Cbll gran* cerebellar granular neurons; *Cbll WM* cerebellar white matter; *Cx* cortex; *DG* hippocampal dentate gyrus; *EC* entorhinal cortex; *Fx* frontal cortex; *Fx WM* frontal cortex white matter; *GP* globus pallidus; *Hippo* hippocampus; *Purkinje* Purkinje neurons; *PUT* putamen; *SN* substantia nigra; *Tx* temporal cortex

In neurons, the highest area density of *MAPT* transcripts was found in the frontal and entorhinal cortices (21.7 and 21.3%, respectively), followed by the temporal cortex, hippocampal CA1 region, basal ganglia and other subcortical regions (2.6–7.6%). Purkinje neurons had the lowest area density of *MAPT* transcripts (1.2 ± 0.1%; Fig. 4a). Pooling neurons from all brain regions revealed an overall area density of 7.2 ± 0.5% of *MAPT* transcripts in neurons. In oligodendrocytes, the highest area density of *MAPT* transcripts was found in the frontal white matter (4.4 ± 0.5%) and lowest in the cerebellar white matter (< 1.0%; Fig. 4b). In astrocytes, a similar area density of *MAPT* transcripts was found across all brain regions examined (1.7–4.5%; Fig. 4c). Overall, pooling each cell type from all brain regions revealed that neurons had the highest area density of *MAPT* transcripts (7.2 ± 0.5%; *p* < 0.001), compared with oligodendrocytes (3.1 ± 0.2%) and astrocytes (3.1 ± 0.2%).

**Fig. 5:**
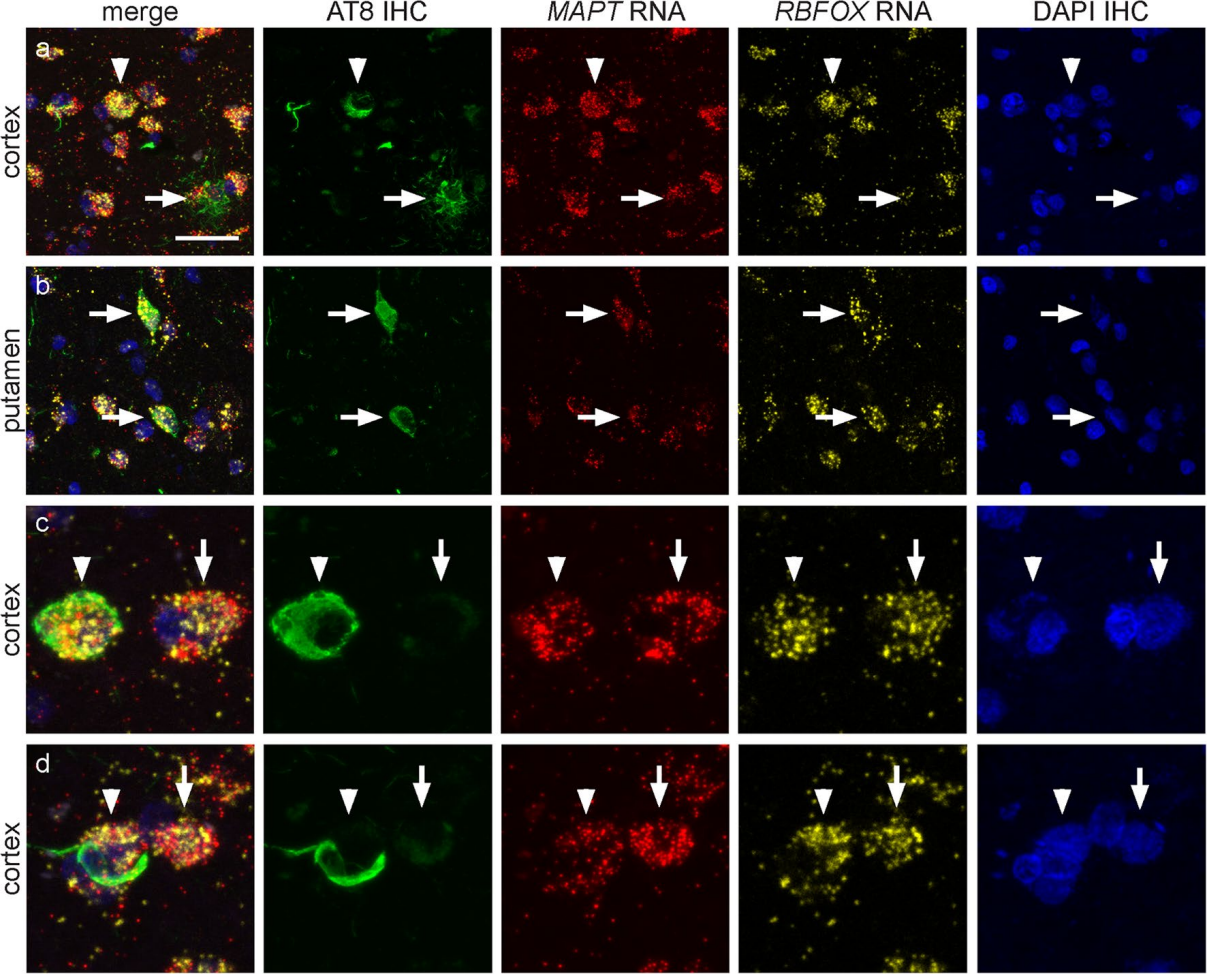
Fluorescent RNAscope combined with phosphorylated tau (AT8) immunohistochemistry in neurofibrillary tangles in progressive supranuclear palsy (PSP). Each horizontal set represents confocal images taken from the same field of view showing AT8-immunostaining (green), *MAPT* transcripts (red) and the neuronal-specific *RBFOX3* (yellow) transcripts, DAPI (blue) and the merge image. **a**, **b** *MAPT* transcripts in neurons containing neurofibrillary tangles (arrowheads) shown in the cortex (**a**) and putamen (**b**). *RBFOX3* transcripts were not observed in astrocytes (arrowhead in (**a**)). **c**, **d** The density of *MAPT* transcripts varied in neurons with (arrowheads) and without (arrows) neurofibrillary tangles. Scale bar in (**a**) represents 30 μm for (**a**), 25 μm for (**b**), and 10 μm for (**c**, **d**)

**Fig. 6:**
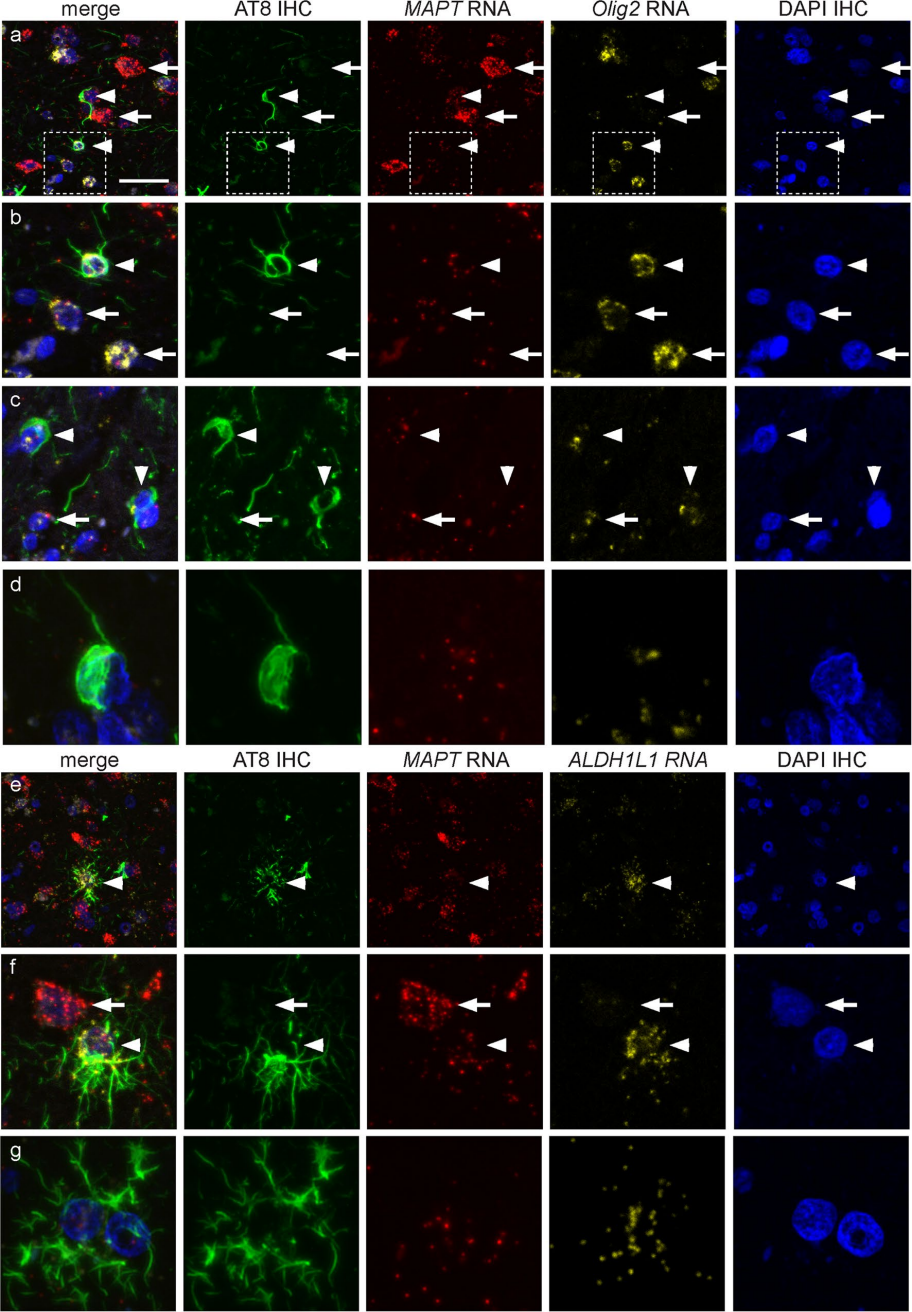
Fluorescent RNAscope combined with phosphorylated tau (AT8) immunohistochemistry in oligodendrocytic and astrocytic inclusions in progressive supranuclear palsy (PSP). Each horizontal set represents confocal images taken from the same field of view showing AT8-immunostaining (green), *MAPT* transcripts (red) and the oligodendrocyte-specific *Olig2* or astrocyte-specific ALDH1L1 (yellow) transcripts, DAPI (blue), and the merge image. **a** Coiled bodies (arrowheads) and neurons (arrow) without neurofibrillary tangles in the frontal cortex. Note the higher density of *MAPT* transcripts in neurons compared with oligodendrocytes. White box in panel (**a**) is enlarged in panel (**b**). **b**, **c** Oligodendrocytes with (arrowheads) and without (arrows) tau-immunopositive inclusions show variation in *MAPT* transcript density, and an enlarged image of an oligodendrocytic coiled body in panel (**d**). **e** Tufted astrocyte (arrowhead) in the frontal cortex. **f**, **g** Enlarged view of tufted astrocytes, which show variation in the density of *MAPT* transcripts. Note the higher density of *MAPT* transcripts in neurons (arrow) compared with the tufted astrocyte (arrowhead) in panel (**f**). Scale bar in (**a**) represents 20 μm for (**a**), 10 μm for (**c**, **d**), 5 μm for (**d**), 30 μm for (**e**), 12 μm for (**f**), and 6 μm for (**g**)

In PSP cases (*n* = 3), the presence of *MAPT* transcripts was confirmed in cells that contained tau-immunopositive inclusions (Fig. 4d–f), including NFTs (Fig. 5a–d), oligodendrocytic coiled bodies (Fig. 6a–d) and tufted astrocytes (Fig. 6e–g). Similar to cells without tau-immunopositive inclusions, the number of *MAPT* transcripts in cells with NFTs, coiled bodies and tufted astrocytes varied.

### Quantification of MAPT transcripts in PSP using fluorescence RNAscope

For each cell type with and without tau-immunopositive inclusions, the pooled area density of *MAPT* transcripts in both the frontal cortex and basal ganglia followed a non-normal distribution pattern, which was confirmed by Kolmogorov–Smirnov test (pooled neurons: *p* < 0.001; pooled NFTs: *p* < 0.001; pooled oligodendrocytes: *p* < 0.001; pooled coiled bodies: *p* < 0.001; pooled astrocytes: *p* < 0.001; pooled tufted astrocytes: *p* < 0.001). Only the *MAPT* transcript area density in NFTs in the frontal cortex showed a normal distribution on Kolmogorov–Smirnov test (*p* = 0.225). Similarly, *MAPT* transcript area density in each cell type with and without tau-immunopositive inclusions followed a non-normal distribution pattern in each region, the frontal cortex and basal ganglia (all *p* < 0.001).

Similar to the control case (Fig. 4), in PSP cases *MAPT* transcript area density differed between cell types without tau-immunopositive inclusions (*p* < 0.001). For *MAPT* transcript area density in cells without tau-immunopositive inclusions, 61% (440/866) of neurons were above the 95% percentile of the Z-score of oligodendrocytic *MAPT* transcript area density and 56% (485/866) of neurons were above the 95% percentile of the *Z*-score of astrocytic *MAPT* transcript area density.

Next, we evaluated nuclear *MAPT* transcript area density in oligodendrocytes and astrocytes without tau-immunopositive inclusions in PSP cases. *MAPT* transcript area density in the nucleus differed between oligodendrocytes and astrocytes (*p* < 0.001). Astrocytes had a higher *MAPT* area density in the nucleus (3.8 ± 0.2%, *n* = 264 cells from 3 PSP cases) compared with oligodendrocytes (2.6 ± 0.1%, *n* = 333 cells from 3 PSP cases).

**Fig. 7:**
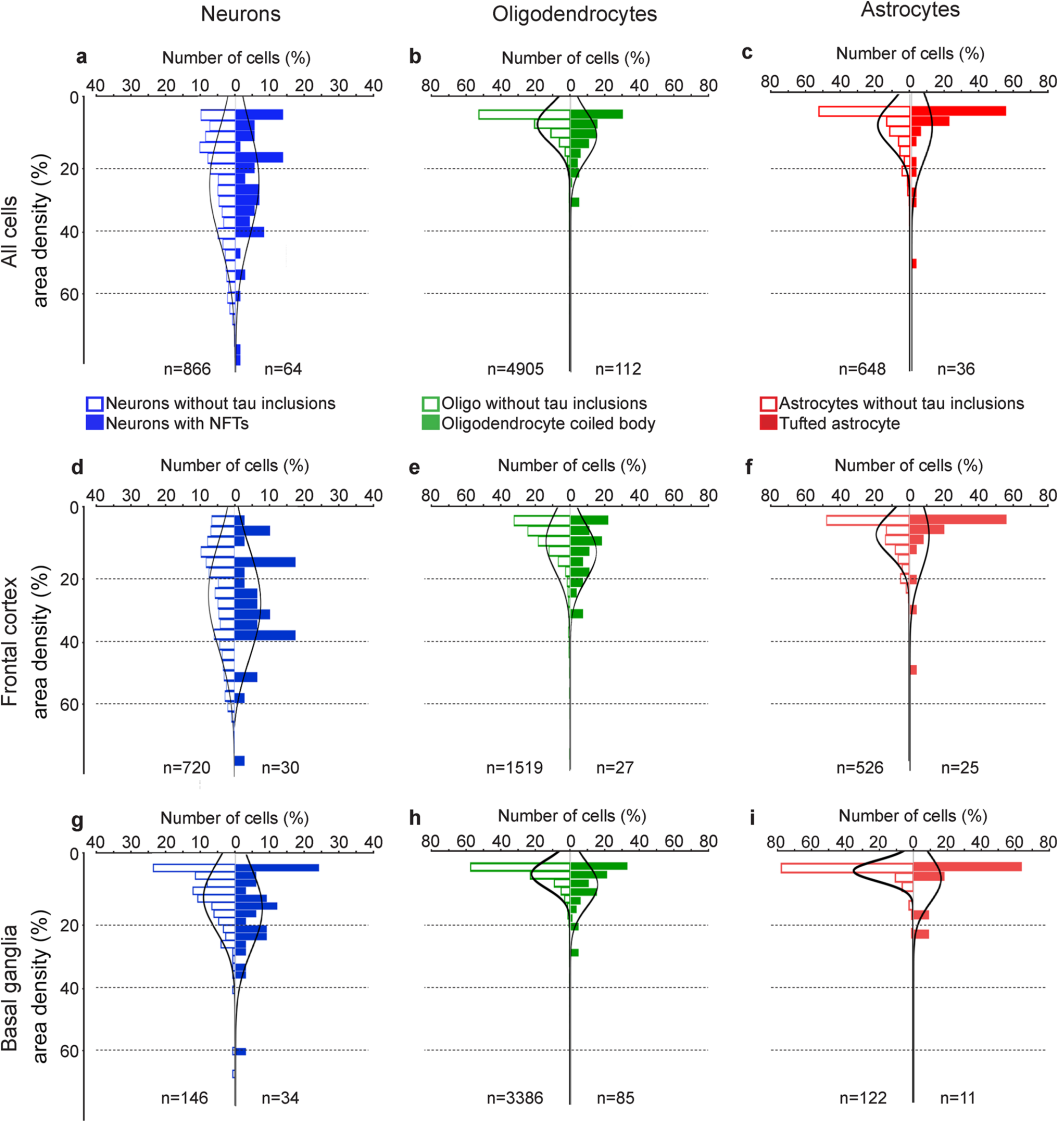
Distribution of area density of *MAPT* transcripts in neurons, oligodendrocytes and astrocytes with and without tau-immunopositive inclusions in PSP. The number of cells (neurons, oligodendrocytes and astrocytes) and area density of *MAPT* transcripts in cells with and without tau-immunopositive inclusions are expressed as the percentage (%). The number of cells analysed are indicated at the bottom of each panel. Panels **a-c** represent the total number of cells analysed in both the frontal cortex and basal ganglia for PSP cases (n = 3), panels **d-f** and **g-i** represent cells analysed in the frontal cortex and basal ganglia, respectively. Blue, green and red outlined bars represent neurons, oligodendrocytes and astrocytes, respectively, without tau-immunopositive inclusions. Solid blue, green and red bars represent neurons with neurofibrillary tangles, oligodendrocytic coiled bodies and tufted astrocytes, respectively. Note the x-axis scale is up to 40% in the neuronal panels, and up to 80% in both oligodendrocyte and astrocyte panels

In PSP cases, the area density of *MAPT* transcripts was compared in neurons, oligodendrocytes and astrocytes with and without tau-immunopositive inclusions within cases, and between brain regions (Figs. 4 and 7). Due to the large variation in area density of *MAPT* transcripts in all cell types with and without tau-immunopositive inclusions, the number of neurons, oligodendrocytes and astrocytes with tau-immunopositive inclusions that were above the z-score (95% percentile) of neurons, oligodendrocytes and astrocytes without tau-immunopositive inclusions was also determined. There was a similar *MAPT* area density in neurons without tau-immunopositive inclusions and those containing NFTs. Higher *MAPT* area  density was observed in NFT-containing neurons (*p* < 0.001) in the basal ganglia in one PSP case only. For *MAPT* area density, only 5% (3/36) of neurons with NFTs were above the 95% percentile *Z*-score of neurons without tau-immunopositive inclusions. Comparing between brain regions, only one NFT-containing neuron in the frontal cortex (1/30, 3%) and three neurofibrillary-containing neurons in the basal ganglia (3/34, 9%) were above the 95% percentile *Z*-score cutoff of *MAPT* transcript area density in neurons without tau-immunopositive inclusions.

Overall, the area density of *MAPT* transcripts in coiled bodies was increased compared with oligodendrocytes without tau-immunopositive inclusions. In the frontal cortex, higher area density of *MAPT* transcripts was observed in coiled bodies in one case (*p* = 0.02), and in the basal ganglia, higher *MAPT* area density was observed in coiled bodies in all cases (Case 1 *p* = 0.002, Cases 2 and 3 *p* < 0.001) compared with oligodendrocytes without tau-immunopositive inclusions. Pooling data for all cases (*p* < 0.001) and for each brain region (frontal cortex *p* = 0.002; basal ganglia *p* < 0.001) showed an increase in area density of *MAPT* transcripts in coiled bodies. However, due to the large variation in *MAPT* area density in all oligodendrocytes, the number of coiled bodies that were above the *Z*-score cutoff (95% percentile) of oligodendrocytes without tau-immunopositive inclusions was determined. For *MAPT* area density in oligodendrocytes, 16% (18/112) of coiled bodies were above the *Z*-score of oligodendrocytes without tau-immunopositive inclusions. Comparing between brain regions, 11% (3/27) and 20% (17/85) of coiled bodies in the frontal cortex and basal ganglia, respectively, were above the *Z*-score cutoff of *MAPT* area density of oligodendrocytes without tau-immunopositive inclusions.

Overall, the *MAPT* transcript area density in tufted astrocytes varied and was increased in the frontal cortex in two cases (Case 1 *p* = 0.005, Case 2 *p* = 0.001), and in one case in the basal ganglia (*p* = 0.02). However, similar *MAPT* transcript area density in astrocytes with and without tau-immunopositive astrocytes was found between brain regions. For *MAPT* transcript area density in astrocytes, 13% (5/36) tufted astrocytes were above the 95% percentile *Z*-score of astrocytes without tau-immunopositive inclusions. Comparing between brain regions, 12% (3/25) and 18% (2/11) of tufted astrocytes in the frontal cortex and basal ganglia, respectively, were above the 95% percentile *Z*-score cutoff of *MAPT* area density of astrocytes without tau-immunopositive inclusions.

### Regional MAPT gene expression in CNS cell populations

**Fig. 8:**
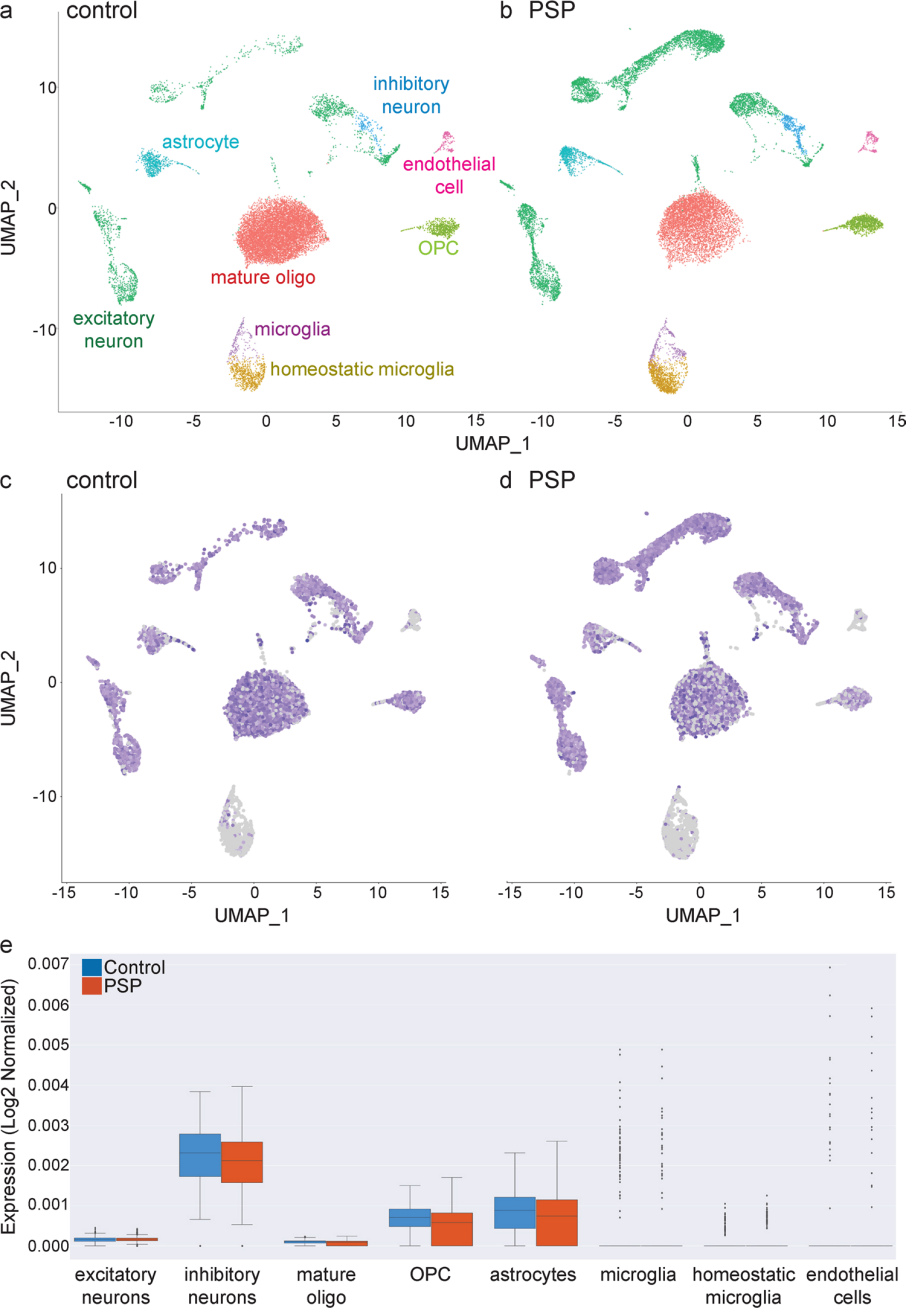
Cell clustering and *MAPT* expression across cell types in control and PSP samples. **a**, **b** Unbiased clustering of snRNAseq data in control (**a**) and PSP (**b**) cases. UMAP plots of cell clusters with each cell group are coloured individually. The colours of the cell labels in (**a**) also correspond to (**b**), and the cell clusters identifies in (**a**) also correspond to the cell clusters in (**c**, **d**). UMAP projection of 31,000 brain cells included, where each cell is grouped into one of the 10 cellular clusters (distinguished by their colours). Cell types were annotated according to expression of known marker genes; cluster name is indicated in the legend. Oligodendrocytes (red) comprised the largest cell cluster. **c**, **d** *MAPT* expression (purple) in controls (**c**) and PSP (**d**) cases in each cell cluster represented in UMAP plots from panels (**a**) and (**b**). **e** *MAPT* expression in controls (blue) and PSP (red) cases. *MAPT* expression was significantly lower in inhibitory neurons, astrocytes and oligodendrocyte precursor cells (OPCs) in PSP cases compared to controls (*P* < 0.0001 for all, n = 3). Mature oligodendrocytes and excitatory neurons contained similar levels of *MAPT* gene expression in controls and PSP

We performed snRNAseq to obtain the transcriptome of cells isolated from freshly frozen brain samples. 31,936 single-nuclei transcriptome profiles including 15,265 from control and 16,671 from PSP cases were generated, and a median UMI count of 8007 per cell (2998 median genes per cell) and 6374 counts per cell (2894 median genes per cell) was detected, respectively. The libraries were sequenced to 88.8% and 89.2% average saturation in control and PSP samples, respectively. We clustered the control and PSP samples together, and the clusters were annotated according to the expression of known cell type marker genes. For example, oligodendrocytes comprised the largest cell cluster, representing 68% and 35% of all cells in controls and PSP, respectively (Fig. 8a, b).

Similar to the RNAscope, snRNAseq data showed that the *MAPT* gene expression varied between and within cell types in the frontal cortex in control cases (*n* = 3; Fig. 8c, d). The number of cell types captured within each cluster varied and for this reason, *MAPT* gene expression was normalized to the number of cells. The highest *MAPT* gene expression was found in inhibitory neurons, followed by astrocytes and oligodendrocyte precursor cells (OPCs). Low levels of *MAPT* gene expression were found in excitatory neurons and mature oligodendrocytes, and *MAPT* was almost absent from endothelial cells.

### Cell type- specific changes in MAPT gene in PSP brain

Single-cell data revealed that *MAPT* gene expression was less in inhibitory neurons, astrocytes and OPCs in PSP cases (*p* < 0.0001 for all, *n* = 3) than in controls. Mature oligodendrocytes and excitatory neurons contained similar levels of *MAPT* gene expression in controls and PSP (Fig. 8e).

## Discussion

This study mapped *MAPT* mRNA expression and compared it with tau protein pathology on a cellular level in the human brain. Implications of our study can be summarized as follows: (i) we show with different methods that in addition to neurons, *MAPT* mRNA is consistently expressed in oligodendrocytes and astrocytes; (ii) cellular *MAPT* expression varies between and within cell types even in the same anatomical area; and finally, (iii) *MAPT* expression is preserved in neurons and glia containing pathological tau protein aggregates.

Tau is the most frequent neurodegenerative disease-associated protein showing fibrillar aggregates in neurons, astrocytes and oligodendrocytes in a number of neurodegenerative diseases. PSP is one major tauopathy that represents all three cytopathologies. Tau pathology in PSP shows sequential involvement of brain regions involving neurons, astrocytes and oligodendrocytes, each with a distinct pattern of hierarchical involvement [41]. Cell-to-cell spreading is discussed for tau and thought to occur via similar mechanisms for neurons and glia [19, 21, 33, 55]. Since glial cells show fibrillar tau aggregates [17] and *MAPT* expression has been assumed to be absent or barely detectable (http://www.brainrnaseq.org) [23, 71], the origin of tau protein pathology in glial cells has been considered to be a consequence of an uptake of released neuronal disease-associated tau.

**Fig. 9:**
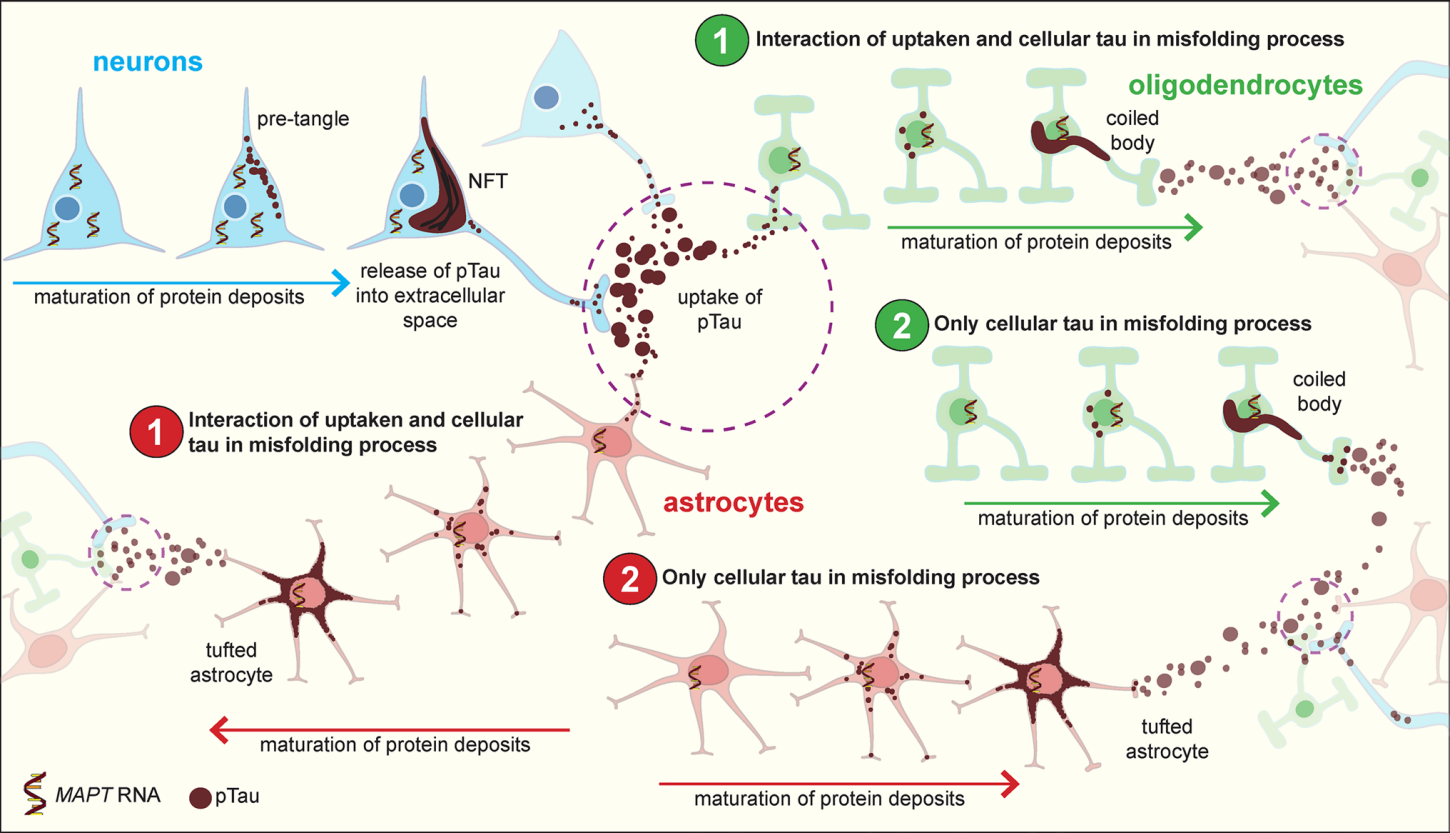
Proposed mechanistic pathways for the cell-to-cell transmission and accumulation of tau-immunopositive inclusions in neurons, oligodendrocytes and astrocytes. Neurons (blue) have a well-described maturation of tau protein deposits, which include early biochemical changes, tau aggregation (pre-tangles) and fibrillization in neurofibrillary tangles (NFT). Neurons contain *MAPT* transcripts, which serve as a cellular pool for increased protein production. In addition, neurons also uptake pTau from the extracellular space, which is released from other neurons containing tau aggregates. This is likely to lead to a facilitated aggregation of tau from both sources: from the local pool for protein production, plus tau uptake from the extracellular space. Similarly, this study shows that both oligodendrocytes (green) and astrocytes (red) contain *MAPT* transcripts, and similar to neurons, this can serve as a local pool for protein production that can be phosphorylated and fibrillized into coiled bodies and tufted astrocytes, respectively. We propose two mechanistic pathways for the accumulation of tau-immunopositive inclusions in oligodendrocytes and astrocytes. In scenario 1, following maturation of protein deposits in neurons, tau is released into the extracellular space, which is taken up by oligodendrocytes and/or astrocytes. Here, there is a facilitated uptake and generation of tau aggregates from two sources; (1) through the uptake of tau, and (2) both oligodendrocytes and astrocytes can draw on their cellular *MAPT* pool for protein production, which can be phosphorylated and fibrillized into coiled bodies and tufted astrocytes, respectively. In scenario 2, oligodendrocytes and astrocytes solely rely on their cellular *MAPT* pool for protein production and following a neurodegenerative event, can be phosphorylated and fibrillized into a coiled body or tufted astrocyte, respectively. Pathological tau in oligodendrocytes and astrocytes can also be released for subsequent uptake by both neurons and other glial cells, thus further accentuating the disease process. Both scenarios may occur at the same time and in the same brain region

Neurons have a well-described maturation process of tau protein deposits, which include early biochemical changes, tau aggregation (pre-tangles), fibrillization in ubiquitinated neurofibrillary tangles (NFTs) and eventually extracellular (ghost) NFTs once the neuron has degenerated [4]. A similar process has been suggested for astrocytic tau inclusions [38]. Neurons contain *MAPT* transcripts, which serve as a local pool for increased protein production. In addition, neurons also uptake misfolded tau from the extracellular space, which is released as tau seeds from surrounding and/or synaptically and functionally interconnected neurons containing misfolded tau aggregates [21]. This is likely to lead to a facilitated aggregation of misfolded tau from both sources. Based on literature data and our observations, we propose two mechanistic scenarios for cell-to-cell transmission and accumulation of tau-immunopositive inclusions in oligodendrocytes and astrocytes leading to hierarchical distribution patterns of pathology (Fig. 9). In both scenarios, the constantly available cellular pool of tau allows it to reach a critical concentration to initiate or facilitate an aggregation process [66]. In scenario 1, following maturation of protein deposits in neurons, tau seeds released into the extracellular space are taken up by oligodendrocytes and/or astrocytes via a variety of yet unidentified mechanisms. Astrocytes have been shown to express phagocytic receptors [7, 9]. Once internalized, tau seeds encounter (i) endogenous cellular mechanisms aiming to remove misfolded tau or (ii) monomeric tau, which feed the seeding of ingested disease-associated misfolded tau. In addition, and eventually independently from ingested misfolded tau seeds (scenario 2), both oligodendrocytes and astrocytes draw on their cellular *MAPT* pool for protein production, which can be phosphorylated, misfolded and aggregated into fibrillar, ubiquitinated and argyrophilic coiled bodies and tufted astrocytes, respectively. Pathological tau in oligodendrocytes and astrocytes can also be released for subsequent uptake by both neurons and other glial cells, further accentuating the disease process. Both scenarios potentially occur at the same time and in the same brain region, but scenario 2 may explain reports describing the presence of oligodendrocytic and astrocytic tau-immunopositive inclusions in the absence of neuronal tau-immunopositive inclusions [1, 36, 37, 39, 40, 44]. This notion is further supported by observations that glial tau pathology shows distinct immunoreactivity patterns of phosphorylated tau isoforms compared to neuronal tau pathology [14, 69]. On the other hand, the possibility that glial cells showing tau pathology in areas lacking neuronal tau pathology ingest misfolded tau from synapses of projecting neurons from another brain region should be also considered [35].

This study shows that *MAPT* RNA transcripts are increased in oligodendrocytic coiled bodies, which was most pronounced in the basal ganglia, an area affected at the earliest disease stages of PSP [41]. In contrast, neurons and astrocytes contained similar, or in some regions, elevated amounts of *MAPT* transcripts in cells with and without tau-immunopositive inclusions. This suggests *MAPT* expression is preserved in these cells with tau-immunopositive inclusions, which is sufficient for the cell to draw upon to preserve normal physiological functioning. Furthermore, this data indicates that accumulation of tau and tau-immunopositive inclusions have no negative feedback effect on *MAPT* expression. However, at some point during the maturation of protein aggregation process for neurons, the neuron will eventually degenerate, as shown by extracellular tangles. Whether a similar degenerative process occurs for tufted astrocytes or whether astrocytes and their physiological machinery are better able to handle fibrillar tau inclusions is yet to be established. The increased amount of *MAPT* transcripts in oligodendrocytes can be interpreted as follows: (i) the coiled body, which comprises fibrillar and insoluble tau, alters the physiological machinery and functioning of the cell; (ii) the oligodendrocyte recognizes the misfolded tau as abnormal, and in response increases *MAPT* expression in an attempt to preserve physiological functioning and maintain the production of physiological tau protein. However, the larger cellular pool of *MAPT* and eventually tau protein that is available can also potentially “feed” the misfolded tau to keep generating/seeding misfolded tau protein and accentuate the pathological process. Alternatively, the increased *MAPT* expression is not necessarily transcribed into protein. However, one study showed similar trends between *MAPT* gene and total tau protein expression between brain regions [65]. However, the possibility that accumulation of misfolded tau could have a direct effect on tau transcription in oligodendroglia and this may be impacted by the ability of the cell to degrade tau protein could also be considered. There is currently limited research on how oligodendrocytes are involved in the pathogenesis of PSP and other tau-depositing disorders, even though the diversity of these inclusions is a major feature in the diagnosis and differentiation of these disorders [13]. Oligodendrocytes have important support functions to neurons, and similar to neurons, have an extensive microtubule network indicating that tau in oligodendrocytes might have functions in regulating microtubule-dependent processes. Preclinical models expressing human tau exclusively in oligodendrocytes showed that oligodendrocytic tau accumulation causes disruption of cellular pathways with detrimental effects on their function [15, 25] that eventually lead to a lack of neuronal support and potentially facilitating neurodegeneration.

We observed a dramatic variation in *MAPT* transcripts between and within cell types even in the same anatomical area, which was also confirmed with snRNAseq showing a wide range of *MAPT* expression in the frontal cortex of controls and PSP patients. The variation in *MAPT* expression within and between cells, and between brain regions, has also been reported using total RNA extracted from human [65] and mouse [66] tissue. This variation likely reflects a dynamic process in the human brain, as transcription at the cellular level is constantly changing in response to physiological or pathogenic demands. As expected, *MAPT* expression was highest in neurons, and results from the snRNAseq study found higher *MAPT* expression in inhibitory neurons compared to excitatory neurons and also in astrocytes compared to oligodendrocytes. These results are in line with recent *MAPT* snRNAseq observations on the entorhinal cortex of ageing cynomolgus monkey [43]. As a limitation, our RNAscope study did not differentiate between inhibitory and excitatory neurons, which is likely to account for some of the variation in *MAPT* expression observed. In addition, since similar *MAPT* transcript area density in both oligodendrocytes and astrocytes was found with tissue-based RNAscope, which was in contrast to that in snRNAseq, we compared the nuclear area density of *MAPT* transcripts. This analysis showed higher area density of *MAPT* transcripts in astrocyte nuclei compared to oligodendrocytes, which aligned with the snRNAseq results. This suggests that oligodendrocytes contain a higher density of *MAPT* transcripts in their cytoplasm. In addition, snRNAseq showed decreased *MAPT* expression in inhibitory neurons, OPCs and astroglia in PSP cases compared with controls, indicating that *MAPT* expression is altered in PSP; we are currently exploring how this contributes to the pathogenesis of disease. However, unlike the RNAscope, the snRNAseq study is not able to evaluate cells with and without tau-immunopositive inclusions. Different methods have been used to evaluate *MAPT* mRNA expression in human tissue including northern blot, in situ hybridization [22, 23] and RT-PCR using total RNA extracted from human brain tissue [24, 29, 65, 66]. In addition, a website indicating lack of *MAPT* expression in astrocytes (http://www.brainrnaseq.org) refers to an article [71] using an immunopanning technique and HepaCAM purified astrocytes from human brain tissue obtained from surgeries for treating epilepsy and tumours and using RNAseq. Our study highlights the differences in the *MAPT* transcription using different methods and shows that the nuclear mRNA expression can be different from cytoplasmic or whole cell mRNA. This has been demonstrated for other genes in the cortex [3] and other tissues [70, 72]. Although our study is not able to demonstrate the level to which the nuclear or cytoplasmic *MAPT* is transcribed to protein expression, it is interesting to observe nuclear *MAPT* transcripts in all three cell types in the light of recent studies highlighting the underappreciated role of nuclear tau in the pathogenesis of tauopathies [2, 42]. The strength of our study is that we evaluated a large number of cells and combined the evaluation of protein pathology and *MAPT* transcripts in the same cells. However, a limitation of this study was that only a small number of control and PSP cases were evaluated and we did not evaluate sex differences in *MAPT* transcript expression.

A study evaluating the overall brain levels of tau protein and tau mRNA (RT and quantitative PCR) as well as 4R-tau and 3R-tau mRNA levels in the frontal cortex in various main tauopathies revealed decreased levels of tau mRNA with an increase in the 4R tau/3R tau mRNA ratio, but preserved tau protein expression in the frontal cortex in PSP brains [29]. Using a tissue-based approach, we complement the observation of maintained tau protein expression in brain homogenates [29, 65] by showing that accumulation of disease-associated tau protein inclusions is not associated with decreased *MAPT* transcripts. In accordance with our observations, recent studies confirm that tau accumulation is not a problem at the transcriptional level, because the expression is relatively constant at different points during ageing [66]. Regulation of *MAPT* expression and splicing is complex [6] and likely different in distinct conditions, and is influenced also by the H1/H2 *MAPT* haplotype [64, 65]. Our study is not able to address how this process is affected during PSP pathogenesis.

Clinical studies and trials are focusing on therapies targeting tau for PSP and two main strategies have been proposed, which are focused on the elimination of pathological tau aggregates or reducing *MAPT* expression with ASO therapies to reduce the amount of tau protein transcribed to prevent its aggregation [5]. However, whether these anti-tau strategies need further consideration is still debated [12, 27]. Our study indicates that the preserved *MAPT* expression in cells accumulating tau protein inclusions might lead to the feeding of the seeding of misfolded tau by constant production of physiological tau. Therefore, a dual-hit strategy could be considered combining the reduction of *MAPT* transcription together with targeting the elimination of misfolded tau. Furthermore, since tau cytopathology does not compromise *MAPT* expression in PSP, and supported by studies on an RNA and protein level using brain homogenates [29, 65, 66], a profound reduction or complete loss of tau protein expression (proteopenia) as a pathogenic component is less likely, while continuous feeding of cellular tau seeding by physiological tau administered for therapeutic reasons might even accentuate the pathological process.

## Conclusions

In PSP and other tau-related neurodegenerative diseases, there is active debate as to whether there is uptake and internalization of tau by glial cells, or whether glia can build fibrillar tau inclusions independently, without an external source. How and why tau accumulates in oligodendrocytes and astrocytes in PSP is not entirely clear, but our study highlights two potential mechanistic pathways for the cell-to-cell transmission and accumulation of misfolded tau protein. Confirming the presence of *MAPT* expression in astrocytes and oligodendrocytes is de facto providing the foundation for tau spreading processes where the uptake seed requires the presence of cell-endogenous tau to be templated into disease specific aggregates [60]. Our observations fine-tune our knowledge on cellular *MAPT* expression by suggesting that nuclear and cytoplasmic RNA expression of *MAPT* might be distinct and opens new avenues for the interpretation of the function of *MAPT*. Importantly, our study provides a rationale for a dual-hit approach to therapies targeting tau aggregation and reduced *MAPT* transcription. Finally, we highlight novel and previously unexplained aspects of disease pathogenesis that will be relevant for therapy development, basic researchers working on cellular mechanisms of tau-related diseases and opens new avenues for neuropathologists working with human tissue.

## Supplementary Information

Below is the link to the electronic supplementary material.

Supplementary file1 (PDF 2443 KB)

## Data Availability

The annonymised datasets generated during and/or analysed during the current study will be made available from the corresponding author on reasonable request from any qualified investigator.
