# Supplementary material for: Cell-specific MAPT gene expression is preserved in neuronal and glial tau cytopathologies in progressive supranuclear palsy
Source: Acta Neuropathol. 2023 Jun 24;146(3):395–414. doi: 10.1007/s00401-023-02604-x (PMC10412651; doi:10.1007/s00401-023-02604-x)

Online Resources

Cell-specific *MAPT* gene expression is preserved in neuronal and glial tau cytopathologies in Progressive Supranuclear Palsy

Shelley L. Forrest[1,2,3], Seojin Lee[1], Nasna Nassir[4], Ivan Martinez-Valbuena[1], Valerie Sackmann[1], Jun Li[1], Awab Ahmed[4], Maria Carmela Tartaglia[1,5], Lars M. Ittner[2], Anthony E. Lang[6], Mohammed Uddin[4,7], Gabor G. Kovacs[1,2,3,6,8]

[1]Tanz Centre for Research in Neurodegenerative Disease, University of Toronto, Toronto, ON, Canada.

[2]Dementia Research Centre, Macquarie Medical School, Faculty of Medicine, Health and Human Sciences, Macquarie University, Sydney, Australia.

[3]Laboratory Medicine Program & Krembil Brain Institute, University Health Network, Toronto, ON, Canada.

[4]College of Medicine, Mohammed Bin Rashid University of Medicine and Health Sciences, Dubai, UAE.

[5]University Health Network Memory Clinic, Krembil Brain Institute, Toronto, ON, Canada.

[6]Edmond J. Safra Program in Parkinson's Disease, Rossy PSP Centre and the Morton and Gloria Shulman Movement Disorders Clinic, Toronto Western Hospital, Toronto, ON, Canada.

[7]Cellular Intelligence (Ci) Lab, GenomeArc Inc., Toronto, ON, Canada.

[8]Department of Laboratory Medicine and Pathobiology and Department of Medicine, University of Toronto, Toronto Ontario, Canada.

Correspondence: Gabor G. Kovacs MD PhD FRCPC. University of Toronto, Tanz Centre for Research in Neurodegenerative Disease (CRND), Krembil Discovery Tower, 60 Leonard Ave Toronto On, M5T 0S8, Canada; Tel: +1 (416) 507-6858.

Email: gabor.kovacs@uhnresearch.ca

Online Resource 1. *MAPT* transcripts in the oculomotor nerve. Each horizontal set represents confocal images taken from the same field of view showing *MAPT* transcripts (red) and the oligodendrocyte-specific *Olig2* (yellow) transcripts, DAPI (blue), and the merge image. **a.** *MAPT* transcripts were not observed in the extraneural segment of the oculomotor nerve. **b.** Occasional *MAPT* transcripts were observed in the axons of the oculomotor nerve. Scale bar in (a) represents 30 μm, applies to both panels.

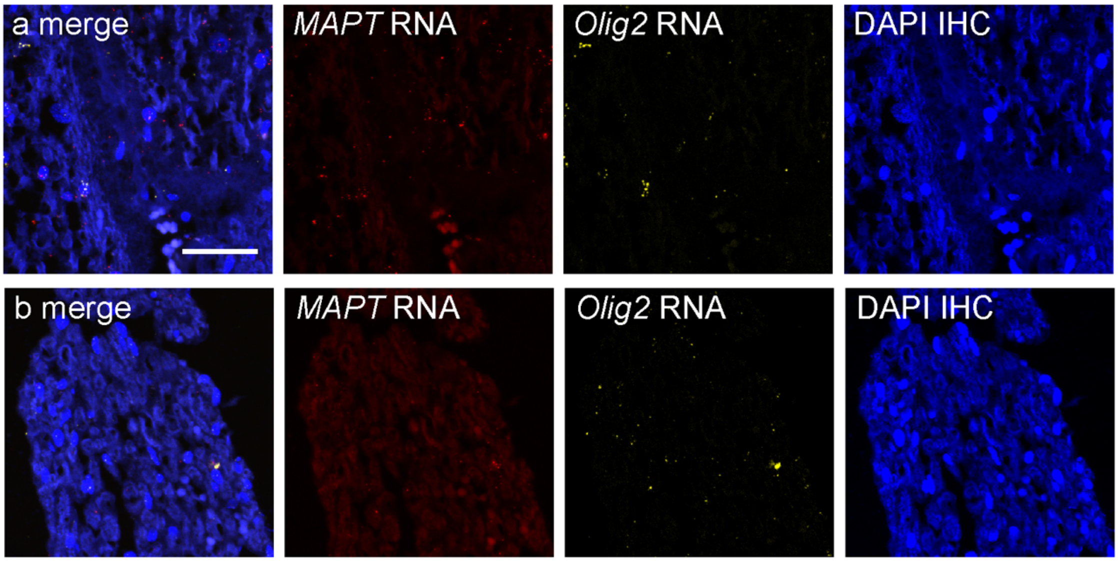

Online Resource 2. *MAPT* transcripts are absent in endothelial cells. Each horizontal set represents confocal images taken from the same field of view showing *MAPT* transcripts (red) and the neuronal-specific *RBFOX3*, oligodendrocyte-specific *Olig2* or astrocyte-specific *ALDH1L1* (yellow) transcripts, DAPI (blue), and the merge image. **a-c.** *MAPT*, *RBFOX3* (a), *Olig2* (b) and *ALDH1L1* (c) transcripts were not observed in endothelial cells (arrowheads). Scale bar in (a) represents 8 µm, applies to all panels.

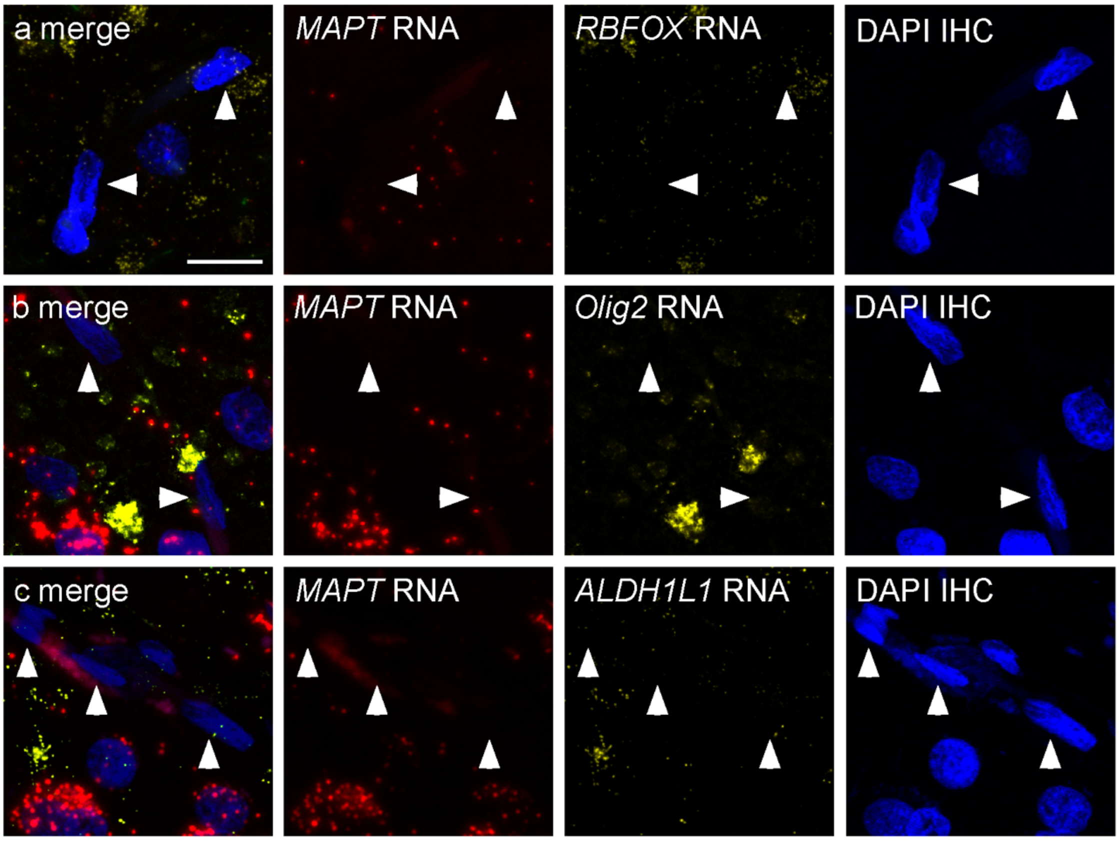

Supplement: Supplementary file 1 — Supplementary file1 (PDF 2443 KB) [file 401_2023_2604_MOESM1_ESM.pdf]
